# Perioperative care of congenital adrenal hyperplasia – a disparity of physician practices in Canada

**DOI:** 10.1186/s13633-018-0063-4

**Published:** 2018-09-10

**Authors:** Munier A. Nour, Hardave Gill, Prosanta Mondal, Mark Inman, Kristine Urmson

**Affiliations:** 1 Division of Pediatric Endocrinology, Department of Pediatrics, College of Medicine, University of Saskatchewan, Royal University Hospital, 103 Hospital Drive, Saskatoon, SK S7N 0W8 Canada; 2 Department of Anesthesiology, Perioperative Medicine and Pain Management, University of Saskatchewan, Saskatoon, Canada; 3 Clinical Research Support Unit, College of Medicine, University of Saskatchewan, Saskatoon, Canada

**Keywords:** Congenital adrenal hyperplasia, Adrenal insufficiency, Anesthesia, Glucocorticoid, Perioperative care

## Abstract

**Background:**

Congenital adrenal hyperplasia (CAH) due to 21-hydroxylase deficiency is the most common cause of primary adrenal insufficiency in children. Current guidelines recommend the use of perioperative stress dose (supraphysiologic) glucocorticoids for children with CAH undergoing anesthesia, although a perceived difference in practice patterns among Canadian pediatric subspecialists prompted an assessment of perioperative glucocorticoid administration.

**Methods:**

We performed a cross-sectional survey of Canadian Pediatric Anesthesia Society (CPAS) and Canadian Pediatric Endocrine Group (CPEG) members via membership email lists to assess reported practice patterns to select clinical scenarios.

**Results:**

Responses were collected from 49 anesthesiologists and 37 pediatric endocrinologists. Less than half of anesthesiologists reported they would provide stress dose corticosteroids for patients undergoing cystoscopy while a significant majority of pediatric endocrinologists reported they would recommend stress dose corticosteroid administration (45% vs 92% respectively, *p* < 0.0001). Twenty-one percent of anesthesiologists reported they would not provide stress dose corticosteroids for patients undergoing laparotomy. Pediatric endocrinologists reported they were more likely to refer to guidelines for management of stress dose steroids (84% vs 51%, *p* < 0.001), with many Canadian pediatric endocrinologists reporting to use institution specific guidelines.

**Conclusions:**

Our results demonstrate a clear difference in the reported approach to perioperative stress dose steroids between pediatric anesthesiologists and pediatric endocrinologists which may impact patient care. Further dialogue is required to address this apparent discrepancy in practice patterns and future research is needed to provide evidence-based practice recommendations.

**Electronic supplementary material:**

The online version of this article (10.1186/s13633-018-0063-4) contains supplementary material, which is available to authorized users.

## Background

Adrenal insufficiency due to classical 21-hydroxylase (21-OH) deficiency congenital adrenal hyperplasia (CAH) requires lifelong therapy with glucocorticoids and usually mineralocorticoids. The purpose of glucocorticoid therapy in 21-OH deficiency CAH is two-fold: to replace for the glucocorticoid deficiency and to suppress excess endogenous androgen production. While most conditions associated with adrenal insufficiency are treated with doses to approximate physiologic replacement [1], classic 21-OH deficiency CAH often requires mild supra-physiologic dosing (10–20 mg/m^2^/day of hydrocortisone equivalent) to prevent virilisation by androgen excess and reduce the risk of adrenal crisis [2, 3].

Current practice guidelines for the treatment of primary adrenal insufficiency advocate for the use of stress dose supraphysiologic corticosteroid treatment during intercurrent illness and perioperatively for both minor and major surgical stress [1] with recommendations for CAH mirroring those for adrenal insufficiency [3]. Despite accurate diagnosis and optimal treatment, children with CAH have been found to have a mortality rate 3 times greater than unaffected children [4].

Currently, there remains debate among some clinicians as to whether perioperative stress-dose supraphysiologic glucocorticoids are required for all surgical procedures and general anesthesia. This includes debate as to replacement for what types of surgery, the optimal timing of administration, and the frequency of dosing. A Cochrane review of adult adrenal insufficiency studies, due to small numbers, was ‘unable to support or refute the use of supplemental perioperative steroids for patients with adrenal insufficiency during surgery [5].’ Even less evidence exists in the pediatric population. Advocates for the use of perioperative ‘stress dose’ steroids cite the potential catastrophic harms of adrenal crisis while those against cite side effect accumulation from repeated treatment with high dose glucocorticoids and the lack of proven benefit – particularly for minor procedures.

The purpose of this study was to compare and contrast practice patterns and beliefs of pediatric anesthesiologists and pediatric endocrinologists in Canada regarding perioperative corticosteroid management in patients with CAH.

## Methods

Local research ethics board approval was obtained for surveys to both anesthesia and endocrinology. An electronic cross-sectional survey was distributed via the Canadian Pediatric Anesthesia Society (CPAS) members’ email list and via the Canadian Pediatric Endocrinology Group’s (CPEG) email list (See Additional files 1 and 2 for anesthesia and endocrine surveys, respectively). The initial email invitation was followed by two additional invitations to complete the survey.

Response frequencies with proportions were calculated for the sample as a whole and by professional group. Responses to questions answered by both groups were compared using the Chi-square test or Fisher’s exact test, the latter when expected cell sizes were less than 5. Alpha was set at 0.05. Analysis was undertaken using SAS, version 9.4 (SAS Institute Inc., Cary, NC, USA).

## Results

We received 49 responses from anesthesiology and 37 responses from pediatric endocrinology. Though definitive recipient denominators could not be ascertained, respective listserv managers approximated 300 anesthesiology members (~ 16.3% response rate) and 85 pediatric endocrinology members (~ 43.5% response rate).

Anesthesia respondents were predominantly male (64%), worked in a university/teaching site (86%), had over 15 years’ experience (61%), and worked in a predominantly pediatric practice (83%). Ninety-two percent of anesthesiologists reported to see less than 6 patients with CAH per year.

Endocrinology respondents were predominantly female (76%), worked in a university/teaching site (92%), and reported predominant ranges of experience of either less than 5 years or greater than 15 years (39 and 36%, respectively).

Endocrinologist were more likely to report being aware of and report to following guidelines for the perioperative management of primary adrenal insufficiency compared with anesthesiologists (84% vs 51%, respectively; *p* = 0.001) with 68% of endocrinologists who used guidelines reported that they use locally established guidelines (Table 1).

**Table 1:** Overall frequencies and comparison of characteristics between pediatric endocrinologists and anesthesiologists

Demographics, n (%)	Combined (*N* = 86)	Anesthesiologists (*n* = 49, 57%)	Endocrinologists (*n* = 37, 43%)	*P*-value^a^
Age				
25–35 years	10 (11.6)	1 (2.0)	9 (24.3)	0.002
36–50 years	41 (47.7)	23 (46.9)	18 (48.7)	
> 50 years	35 (40.7)	25 (51.0)	10 (27.0)	
Sex				
Male	39 (46.4)	30 (63.8)	9 (24.3)	0.0003
Female	45 (53.6)	17 (36.2)	28 (75.7)	
Practice characteristics, n (%)				
Practice type				
University/ teaching	76 (88.4)	42 (85.7)	34 (91.9)	0.50
Other	10 (11.6)	7 (14.3)	3 (8.1)	
Years in practice				
< 5	13 (15.9)	0	13 (39.4)	< 0.0001
5–10	17 (20.7)	12 (24.5)	5 (15.1)	
11–15	10 (12.2)	7 (14.3)	3 (9.1)	
> 15	42 (51.2)	30 (61.2)	12 (36.4)	
Congenital adrenal hyperplasia in practice				
Administer stress dose steroids for cystoscopy				
Yes	55 (65.5)	21 (44.7)	34 (91.9)	< 0.0001
No	29 (34.5)	26 (55.3)	3 (8.1)	
Concerned about repeated single high dose steroids				
Yes	22 (26.2)	17 (36.2)	5 (13.5)	0.019
No	62 (73.8)	30 (63.8)	32 (86.5)	
Consult opposite specialty				
Always	20 (23.3)	14 (28.6)	6 (16.2)	0.16
Frequently	18 (20.9)	12 (24.5)	6 (16.2)	
Occasionally	25 (29.1)	14 (28.6)	11 (29.7)	
Never	23 (26.7)	9 (18.4)	14 (37.8)	
See endocrinology before surgery				
Sometimes	8 (9.5)	5 (10.6)	3 (8.1)	0.99
Yes	76 (90.5)	42 (89.4)	34 (91.9)	
Follow guidelines for stress dose decision				
Yes	56 (65.1)	25 (51.0)	31 (83.8)^b^	0.001
No	30 (34.9)	24 (49.0)	6 (16.2)	

^a^Comparisons by Chi-square test or Fisher’s exact test

^b^Guideline type: Local centre guidelines *n* = 21 (67.7%), published clinical practice guidelines *n* = 7 (22.6%), other *n* = 3 (9.7%). Frequencies in variable categories do not always sum the totals because of missing data

Anesthesiology respondents reported they were less likely to provide stress-dose steroids for a cystoscopy compared with endocrinology respondents (45% vs 92% respectively, *p* < 0.0001). Only 8% of pediatric endocrinologists responded that they would not recommend stress dosing, and instead recommend continuation of daily glucocorticoid replacement (Table 1). Twenty-one percent of anesthesiologists reported they would not follow stress-dose guidelines for patients undergoing laparotomy. More than half of endocrinologists (57%) reported that they would recommend stress dose steroids for all patient with CAH undergoing anesthesia, while the remaining reported they would base recommendations on the type of procedure (13.5%), CAH severity (13.5%) or both (16%) (See Table 2).

**Table 2:** Group-specific responses of anesthesiologists and pediatric endocrinologists regarding perioperative care of pediatric congenital adrenal hyperplasia patients

	Anesthesiologists *n* = 48	Endocrinologists *n* = 37
Congenital adrenal hyperplasia in practice, n (%)		
Number of CAH patients in endocrinology practice		
None		2 (5.4)
1–5		16 (43.2)
6–10		8 (21.6)
11–15		4 (10.8)
> 15		7 (18.9)
Number of pediatric CAH patients in anesthesia practice per year		
≤ 5	44 (91.7)	
6–12	4 (8.3)	
Percentage of practice that involves pediatric anesthesia		
1–25	3 (6.2)	
26–50	5 (10.4)	
51–75	5 (10.4)	
76–100	35 (72.9)	
Pediatric congenital adrenal hyperplasia management, n (%)		
Common to see CAH patients regarding stress dose prior to surgery		
Yes		34 (91.9)
Sometimes		3 (8.1)
Consult another anesthesiologist regarding stress dosing		
Frequently (> 50% of the time)	3 (6.3)	
Occasionally (< 50% of the time)	15 (31.3)	
Never	30 (62.5)	
Frequency of consult to anesthesiologist regarding stress dose		
Always		6 (16.2)
Frequently		6 (16.2)
Occasionally		11 (29.7)
Never		14 (37.8)
Frequency of consult to endocrinologist regarding stress dose		
Always	14 (28.6)	
Frequently	12 (24.5)	
Occasionally	14 (28.6)	
Never	9 (18.4)	
Endocrinology referral common at home institution for any surgery in children with CAH		
Yes	42 (89.4)	
Unsure	5 (10.6)	
Recommend corticosteroid stress dose for children with CAH undergoing anesthesia		
Always		21 (56.8)
Severity dependent		5 (13.5)
Procedure dependent		5 (13.5)
Severity and procedure dependent		6 (16.2)
Minor procedure recommendation/management (e.g. cystoscopy), n (%)		
Recommended dosing:		
Mild stress dosing (20–40 mg/m^2^ of HC equivalent)		26 (70.3)
High dose (50–100 mg/m^2^ of HC equivalent)		8 (21.6)
Baseline therapy		3 (8.1)
Follow guidelines for minor procedures?		
Yes	21 (44.7)	
No	26 (55.3)	
Steroid dosing if typically using dexamethasone for prevention of PONV		
Omit dexamethasone, give stress dose	23 (48.9)	
Give dexamethasone + baseline steroid	12 (25.5)	
Give dexamethasone + stress dose	6 (12.8)	
Other	6 (12.8)	
Major procedure management (e.g. laparotomy), n (%)		
Follow guidelines		
Yes	37 (78.7)	
No	10 (21.3)	

*CAH* congenital adrenal hyperplasia, *HC* Hydrocortisone, *PONV* post-operative nausea and vomiting

Anesthesiologists were more likely than endocrinologists to be concerned about repeated, single high dose steroid exposures in patients with CAH undergoing anesthesia (36% vs. 14%, *p* < 0.02) with many reporting that they felt stress dose guidelines led to overtreatment.

## Discussion

Our survey of Canadian pediatric endocrinologists and pediatric anesthesiologists or anesthesiologists with pediatrics in their practice demonstrates a difference in reported practices regarding perioperative glucocorticoid administration in children with CAH. Pediatric endocrinologists were more likely to recommend glucocorticoid ‘stress dose’ coverage perioperatively for both less and more invasive procedures in children with CAH.

Determining the ideal time, amount and duration of stress dose glucocorticoids for patients with primary adrenal insufficiency is largely unknown and research into this area has been conflicting or absent. Owing to small patient numbers, different glucocorticoid formulations, a variety of underlying causes of adrenal insufficiency and varying degrees of surgical stress, conclusive evidence to provide clear recommendations has been sparse. As a result, current practice guidelines are based more so on expert opinion and may be aimed to safeguard against worst case scenarios [6].

Indications for stress-dose supraphysiologic glucocorticoids in CAH have evolved over recent years, particularly during times of ‘normal’ stress, such as school examinations, emotional stress, or exercise. Studies examining high intensity exercise, for example, have demonstrated that additional supraphysiologic glucocorticoid administration provided no clinically meaningful benefit to hormonal, metabolic, or cardiorespiratory outcomes [7]. In the surgical patient with CAH, the impact of fasting, with its potential for stress response, hypoglycemia and dehydration, has not been studied.

A number of studies have used healthy children to document hormonal responses to anesthesia and surgery. Research by Hsu et al. demonstrated a 3-fold increase in salivary cortisol concentrations in 110 healthy children following surgery or sedation [8]. Cortisol levels were highest in the recovery phase after the procedure was completed; however, levels were not influenced by degree of sedation achieved nor type of procedure performed. Some studies have demonstrated a graded response of cortisol production to the degree of surgical stress [9], while other studies have attributed this to anesthesia reversal and recovery and not the surgical trauma itself [10]. Anesthesia for minimally  invasive procedures and medical imaging has been shown to result in minimal cortisol excursions in healthy children [11, 12]. Taken together, these studies are certainly influenced by medication choices and anesthetic techniques which have changed significantly over recent years yielding significant effects on physiologic response to surgery and anesthesia [13]. Given the heterogeneity in populations studied, methods employed, degrees of surgical intervention, and sedation techniques, it is unclear whether cortisol excursion following anesthesia and surgery is merely a biochemical, physiologic response or whether it is important for patient recovery [8].

Within adult practice, a growing tendency to maintain a patient’s maintenance (physiologic) dose of steroids throughout the surgical period in those with adrenal insufficiency *secondary to exogenous suppression* has elicited much controversy. Recent reviews in adults with adrenal insufficiency secondary to chronic corticosteroid use have suggested that patients do not require stress dose steroids for surgery [14]. The theoretic rationale for this has been that patients with secondary suppression are more likely to demonstrate an endogenous hypothalamic-pituitary-adrenal axis response to severe physiological stresses. However, it is important to note that authors have been careful to point out that these reviews do not apply to causes of primary adrenal insufficiency, such as CAH or Addison’s disease, whereby no endogenous glucocorticoid production is possible, even under marked physiologic stress. Proponents of withholding stress dose steroids in adults with secondary suppression have been careful to clarify this difference and, thus recommend coverage in all patients with primary adrenal insufficiency [14]. Furthermore, cases of adrenal crisis in primary adrenal insufficiency have emerged following the adoption of some of these practices associated with adrenal insufficiency secondary to exogenous suppression [15].

Current Endocrine Society guidelines endorse the use of stress dose (supraphysiologic) steroid graded on the degree of surgical stress (See Table 3) [1]. These recommendations were largely adapted from expert opinion and their intent was not to cover surgical stress per-se, but rather to cover in case of unforeseen complications. As a result, the “dose increase is not intended to mimic the median cortisol increase in healthy subjects during such procedures. Instead, it is intended to mimic the maximum cortisol increase, which may occur in euadrenal subjects triggered during these procedures, potentially induced by some unforeseen events (e.g. postoperative bleeding) [6].” The rationale for prophylactically providing these stress dose (supraphysiologic) glucocorticoids is to pre-emptively prevent clinical deterioration as euadrenal patients would have an immediate response to unforeseen circumstance while adrenal insufficient patients would be delayed until after clinical decompensation, physician recognition, glucocorticoid administration and its onset of action. In our survey, we found that 21% of anesthesiologist respondents reported they would not administer stress dose steroids for those patients undergoing laparotomy. Unfortunately, from our questionnaire, we did not gather the rationale as to why these physicians would omit stress dose treatment in such a scenario.

**Table 3:** Management of pediatric adrenal insufficiency in specific situations. Adapted from [1]

Condition	Suggested Action
Home management of illness with fever. Unable to tolerate oral medications due to gastroenteritis or trauma	Hydrocortisone replacement doses doubled (> 38 °C) or tripled (> 39 °C) until recovery (usually 2 to 3 days); increased consumption of electrolyte containing fluids as tolerated
	IM/SC Hydrocortisone 50 mg/m^2^ or estimate; infants 25 mg, school-aged children 50 mg, adolescents 100 mg.
Minor to moderate surgical stress	Intramuscular/Intravenous Hydrocortisone 50 mg/m^2^ or hydrocortisone replacement doses doubled or tripled
Major Surgical Stress with general anesthesia, trauma, or diseases that require intensive care	Hydrocortisone 50 mg/m2 intravenous followed by hydrocortisone 50–100 mg/m^2^/d divided q6 h
	Weight-appropriate continuous intravenous fluids (dextrose containing)
	Rapid tapering and switch to oral regimen depending on clinical state
Acute adrenal crisis	Rapid bolus of normal saline (0.9%) 20 mL/kg. Can repeat up to a total of 60 mL/kg within 1 h for shock.
	Hydrocortisone 50–100 mg/m2 bolus followed by hydrocortisone 50–100 mg/m^2^/d divided q 6 h
	For hypoglycemia: dextrose 0.5–1 g/kg of dextrose or 2–4 mL/kg of D25W (maximum single dose 25 g) infused slowly at rate of 2 to 3 mL/min. Alternatively, 5–10 mL/kg of D10W for children < 12 y old
	Cardiac monitoring: Rapid tapering and switch to oral regimen depending on clinical state

Given the rationale for treatment recommendations, one may stand to argue whether low-stress procedures with low risk of complications indeed require stress dose (supraphysiologic) prophylactic glucocorticoid doses (i.e. cystoscopy or medical imaging). Over half of the responding anesthesiologists (55%) reported they would not provide supraphysiologic glucocorticoids for these clinical situations, as compared with 8% of endocrinologists. While it is known that long term supraphysiologic steroid administration is associated with many iatrogenic medical complications, the short duration and relatively small (comparative) doses used perioperatively have not been demonstrated to cause harm.

From our data, anesthesiologists were more likely to be concerned about side effects from repeated, single doses of high dose glucocorticoids; however, these concerns merit clinical reflection given that many anesthesiologists prophylactically use dexamethasone for the prevention of post-operative nausea and vomiting at glucocorticoid equivalent doses that are at least as high or greater than those recommended by single dose perioperative stress dose guidelines.

### Limitations

This study relied upon voluntary response from a national digital mail out to the prominent Canadian organizations for pediatric anesthesiology and pediatric endocrinology. As a result, the exact total sample size and thus the proportion of responders is not definite.

## Conclusions

This physician survey was performed to highlight a suspected difference in practices between pediatric anesthesia and pediatric endocrine colleagues ultimately caring for the same patient cohort and to begin a dialogue among specialty groups. While the authors of this paper still advocate for the use of perioperative stress dose glucocorticoid prescription for primary adrenal insufficiency in pediatrics as per the limited guidelines currently available, it must be recognized that available literature is sparse and further study to guide evidence based recommendations are still required. This survey demonstrates a clear discrepancy in practice patterns and highlights the need for further discussion between care providers. The risk of adverse effects from prophylactic stress dose (supraphysiologic) steroids are trivial. While the risks associated with foregoing prophylactic therapy are similarly low, the potential rare outcome in unforeseen circumstances may be catastrophic.

## Additional files

Additional file 1: Survey questions sent to Canadian Pediatric Anesthesia Society (CPAS) members. (DOCX 16 kb)

Additional file 2: Survey questions sent to Canadian Pediatric Endocrine Group (CPEG) members. (DOCX 17 kb)

## Abbreviations

21-OH: 21-hydroxylase
CAH: Congenital adrenal hyperplasia
HC: Hydrocortisone
PONV: Post-operative nausea and vomiting
