# Supplementary material for: Perioperative care of congenital adrenal hyperplasia – a disparity of physician practices in Canada
Source: Int J Pediatr Endocrinol. 2018 Sep 10;2018:8. doi: 10.1186/s13633-018-0063-4 (PMC6131860; doi:10.1186/s13633-018-0063-4)
Supplement: Supplementary file 1 — Survey questions sent to Canadian Pediatric Anesthesia Society (CPAS) members. (DOCX 16 kb) [file 13633_2018_63_MOESM1_ESM.docx]

**Anesthesia Questionnaire**

1. Please choose your gender
   1. Male
   2. Female
2. Please choose your age bracket
   1. 25-35
   2. 36-50
   3. 51-65
   4. Over 65
3. Please choose type of anesthesia practice: - drop down menu
   1. University/Teaching
   2. Community
   3. Combination
   4. Anesthesia Assistant
   5. General Practice Anesthesia
4. How many years have you been in practice?
   1. Less than 5
   2. 5-10 years
   3. 11-15 years
   4. Over 15 years
5. What percentage of your practice involves pediatric anesthesia?
   1. 1-25 %
   2. 26-50 %
   3. 51-75 %
   4. 76-100 %
6. How many pediatric patients would you see with primary adrenal insufficiency in your practice in a year?
   1. More than 12 (once a month)
   2. Between 6-12
   3. Less than 6 per year
7. At your facility is it common practice for children with primary adrenal insufficiency to see an endocrinologist prior to any surgery?
   1. Yes
   2. No
   3. Unsure
8. How often do you consult another anesthesiologist in making decisions regarding peri-operative stress dose steroids for children with primary adrenal insufficiency undergoing anesthesia?
   1. Never
   2. Occasionally (<50% of the time)
   3. Frequently (>50% of the time)
   4. Always
9. How often do you consult an endocrinologist in making decisions regarding peri-operative stress dose steroids for children with primary adrenal insufficiency undergoing anesthesia
   1. Never
   2. Occasionally (<50% of the time)
   3. Frequently (>50% of the time)
   4. Always

The next few questions pertain to congenital adrenal hyperplasia (CAH), one form of primary adrenal insufficiency. CAH is a disorder involving a deficiency of an enzyme involved in the synthesis of cortisol, aldosterone, or both.

1. Are you aware of stress dose steroid guidelines for children with CAH undergoing anesthesia?
   1. Yes
   2. No
2. The Endocrine Society’s Clinical Guidelines on CAH state that patients should have stress-dose steroids for surgery accompanied by general anesthesia in doses of: infants and preschool children-hydrocortisone 25 mg iv, school-aged children hydrocortisone 50 mg iv, adults hydrocortisone 100 mg iv. Would you follow these guidelines for a cystoscopy?
   1. Yes
   2. No (if selected please provide your steroid management strategy)
3. Using the same guidelines, would you follow these guidelines for a laparotomy?
   1. Yes
   2. No
4. If providing dexamethasone (no mineralocorticoid activity) consistent with the Consensus Guidelines for the Management of Postoperative Nausea and Vomiting for a cystoscopy, would you:
   1. Provide dexamethasone as well as stress dose steroids as recommended by the endocrine society: infants and preschool children-hydrocortisone 25 mg iv, school-aged children hydrocortisone 50 mg iv, adults hydrocortisone 100 mg iv
   2. Omit stress dose steroids and continue baseline daily steroid dose
   3. Omit dexamethasone and provide stress dose steroids as per guidelines
   4. Other management strategy. If Other, please specify drug and dose regimen
5. Are you concerned about repeated single high dose steroids in patients with adrenal insufficiency undergoing anesthesia?
   1. Yes
   2. No
6. Please indicate other factors that you think are important in considering peri-operative high dose steroids for patients with adrenal insufficiency undergoing anesthesia:
