# Supplementary material for: Perioperative care of congenital adrenal hyperplasia – a disparity of physician practices in Canada
Source: Int J Pediatr Endocrinol. 2018 Sep 10;2018:8. doi: 10.1186/s13633-018-0063-4 (PMC6131860; doi:10.1186/s13633-018-0063-4)
Supplement: Supplementary file 2 — Survey questions sent to Canadian Pediatric Endocrine Group (CPEG) members. (DOCX 17 kb) [file 13633_2018_63_MOESM2_ESM.docx]

**Endocrine Questionnaire**

**Demographics**

1. Please choose your gender
   1. Male
   2. Female
2. Please choose your age bracket
   1. 25-35 years
   2. 36-50 years
   3. 51-65 years
   4. >65 years
3. In which province do you practice?
   1. British Columbia
   2. Alberta
   3. Saskatchewan
   4. Manitoba
   5. Ontario
   6. Quebec
   7. Nova Scotia
   8. Newfoundland
   9. Other

**Practice Description**

1. Current careers stage
   1. Resident
   2. Fellow
   3. Staff Pediatric Endocrinologist
   4. Other
2. Please choose your type of endocrine practice
   1. University/Teaching
   2. Community
   3. Combination
   4. Other
3. How many years have you been in practice (if post-fellowship)
   1. Less than 5 years
   2. 5-10 years
   3. 11-15 years
   4. Greater than 15 years
   5. Not applicable
   6. Perioperative Management of CAH

**Perioperative Management of CAH**

1. How many patients with 21-OH Deficiency (21OHD) Congenital Adrenal Hyperplasia (CAH) would you estimate to currently follow within your practice?
   1. None
   2. 1-5
   3. 6-10
   4. 10-15
   5. >15
2. Is it common practice for you to see/advise your 21OHD CAH patients regarding corticosteroid dosing prior to surgery?
   1. Yes
   2. No
   3. Sometimes
3. Are there guidelines you use in order to guide your stress dose corticosteroid practices for children with 21OHD CAH undergoing anesthesia?
   1. Yes
   2. No
4. If you answered 'yes' above, what type of guidelines do you refer to?
   1. Published Clinical Practice Guidelines (i.e. Endocrine Society, etc.)
   2. Local Centre Guidelines
   3. Other, please specify
5. Do you recommend stress dose corticosteroids for children with 21OHD CAH undergoing anesthesia?
   1. Never
   2. Always
   3. Procedure dependant
   4. CAH Severity dependent (i.e. Classic, Non-classic, Salt-Wasting, etc.)
   5. Procedure AND CAH severity dependent
6. How often do you consult with an anesthesiologist in making decisions regarding peri-operative stress dose corticosteroids for children with 21OHD CAH undergoing anesthesia?
   1. Never
   2. Occasionally
   3. Frequently
   4. Always
7. For minor procedures, such as cystoscopy, in children with classic 21OHD CAH would you:
   1. Continue baseline steroid therapy as per child's home doses
   2. Recommend 'mild' stress dosing prior to surgery (~20-30 mg/m2/day of hydrocortisone equivalent)
   3. Recommend high dose peri-operative steroids (~50-100 mg/m2/day of hydrocortisone equivalent)
   4. Omit steroids day of surgery and only provide rescue steroids if the patient required them clinically
8. Are you concerned about repeated single high dose steroids in patients with 21OHD CAH undergoing anesthesia?
   1. Yes
   2. No
9. If you answer 'yes' for the question above, please elaborate:
